# Bellevue Place

**Published:** 1867-07

**Authors:** 


### *Bellevue Place.*

We most cheerfully call attention to the notice in our advertising pages of this quiet and pleasant retreat for the unfortunate insane. From the testimonials we have seen, we feel confident in recommending Dr. Patterson as a gentleman of experience and ability in this department of therapeutics. In opening this retreat, Dr. P. will prove himself a benefactor to the public.

